# The generalized predictive control of bacteria concentration in marine lysozyme fermentation process

**DOI:** 10.1002/fsn3.850

**Published:** 2018-10-18

**Authors:** Xianglin Zhu, Ziyan Zhu

**Affiliations:** ^1^ School of Electrical and Information Engineering Jiangsu University Zhenjiang Jiangsu China

**Keywords:** bacteria concentration, generalized predictive control, least squares support vector machine, lysozyme, particle swarm optimization

## Abstract

Due to the high degree of strong coupling and nonlinearity of marine lysozyme fermentation process, it is difficult to accurately model the mechanism. In order to achieve real‐time online measurement and effective control of bacterial concentration during fermentation, a generalized predictive control method based on least squares support vector machines is proposed. The particle swarm optimization least squares support vector machine (PSO‐LS‐SVM) model of lysozyme concentration is established by optimizing the regularization parameters and the kernel parameters of the least squares support vector machine by particle swarm optimization. To avoid the nonlinear problems in predictive control, the model is linearized at each sampling point and the generalized predictive algorithm is used to predict the bacteria concentration of lysozyme. The experimental simulation shows that the least squares support vector machine model with particle swarm optimization can achieve good prediction effect. The linearized model performs generalized predictive control, which makes the total activity of the enzyme increased from 60% to 80% and the yield improved by 30%.

## 1 INTRODUCTION

Due to the characteristics of nonlinear, time‐varying, and high dimension in the process of microbial fermentation, parameter detection is the key to determine the optimal control point in each stage of fermentation process (Zhong, He, Pi, & Sun, 2005). With the limitation of hardware detection technology, the bacteria concentration of marine lysozyme is difficult to measure online in real time and is prone to large errors when effective control is applied (Boulkaibet, Belarbi, Bououden, Marwala, & Chadli, 2017; Wang et al., 2000). Generalized predictive control (GPC) has strong robustness, which is applicable to stochastic systems and online identification. It has the strategies of moving horizon optimization, multi‐step prediction, and feedback compensation in the predictive control algorithm, which means that the feedback compensation control is obtained by optimizing a certain performance index in the moving finite time interval,while the requirements of the mathematical model are higher when improving the accuracy of predictive control. (Araúzo‐Bravo et al., 2004; Yang, Li, Ding, Guo, & Hao, 2012; Zhu, Liu, Sun, & Wang, 2010). The least squares support vector machine (LS‐SVM) replaces the inequality constraint condition in SVM standard algorithm by using equality constraints and overcomes the dimensionality disaster problem of classical quadratic programming method for solving SVM (Li, Su, & Chu, 2007; Liu, Jiang, & Fang, 2008). In the actual lysozyme fermentation process, the fermentation process is a slow time‐varying process, which does not require high real‐time performance because of the physiological characteristics of lysozyme itself. Therefore, although the lag caused by the LS‐SVM‐based prediction model is longer than that caused by the SVM‐based prediction model, it does not affect the bacterial concentration prediction. (Huang, Zhai, Sui, & Chai, 2010; Suykens & Vandewalle, 1999; Wang, Zhen, & Zhu, 2013). However, the regularization parameter *C* and the kernel parameter *σ* of the LS‐SVM model have a great influence on the fitting precision and generalization ability. Particle swarm optimization (PSO) is a population‐based stochastic optimization method, which can simultaneously search for more regions in the solution space of the target function to be optimized, and solves the problem of LS‐SVM parameter selection (Li, Tang, & Liu, 2010; Yan & Cui, 2013). Therefore, this paper proposes a nonlinear model for establishing bacteria concentration after optimization of LS‐SVM using PSO. To avoid solving nonlinear problems in predictive control (Liu, Su, & Zhu, 2004; Mahmoodi, Poshtan, Jahed‐Motlagh, & Montazeri, 2008; Xi, Li, & Lin, 2013), the obtained LS‐SVM nonlinear model is linearized at each sampling point, the generalized predictive control algorithm is used to solve multi‐step prediction and process control is performed on its prediction parameters.

## 2 BACTERIA CONCENTRATION MODELING ANALYSIS

Lysozyme is an important enzyme preparation, which can hydrolyze mucopolysaccharide in pathogenic biomass. According to the bacteriolytic characteristics of lysozyme, it can be used in medical treatment, food preservation, and bioengineering. Especially in food preservation, it has been widely used in aquatic products, meat products, cakes, sake, wine, and beverages to replace chemically synthesized food preservatives (Ren et al., 2013; Wang et al., 2000; Zhao, Bai, Zhang, & Wu, 2010). However, the bacteria concentration is too high or too low, which can make the fermentation broth viscous or dilute, and the poor mass transfer conditions will make the product enzyme difficult to synthesize in the fermentation process. Therefore, reasonable control of bacteria concentration can increase enzyme activity and yield. Through the in‐depth analysis of the process mechanism, the substrate feed rate has a great influence on the bacteria concentration, and the reasonable feed rate can improve the product activity (Huang, Sun, Sun, Liu, & Nie, 2013; Zhu, He, Sun, & Wang, 2013). The lysozyme concentration model can be expressed in the following nonlinear form:

(1) $$y\left(t\right)=f\left(u(t-1),\;u\;(t-2),\cdots \;,u\;(t-m)\;,y\;(t-1),\cdots ,\;y\;(t-n)\right)$$

where *f*(g) represents a complex nonlinear function.

## 3 ESTABLISHMENT OF LS‐SVM MODEL BASED ON PSO OPTIMIZATION

### 3.1 Establishment of LS‐SVM model

There is given a training set {*x*_*i*_, *y*_*i*_} with *N* data, and *x*_*i*_ is input data, *y*_*i*_ is output data, *x*_*i*_ ⊂ *R* ^*n*^, *y*_*i*_ ⊂ *R*,* i* = 1, 2, ···, *N*.

The LS‐SVM model can use the following functions in the eigenspace:

(2) $$y\left(x\right)=w^{T}\phi \left(x\right)+\delta$$

where *ϕ*(•):*R* ^*n*^ → *R* ^*nh*^ is a function that maps the input data of the original space to the higher‐dimensional eigenspace, *w* is weight vector, *δ* is constant deviation, *w* ∊ *R* ^*nh*^, *δ* ∊ *R*.

The LS‐SVM regression optimization problem is as follows:

(3) $$\min_{w,b,e}\;J\left(w,e\right)=\frac{1}{2}w^{T}w+\frac{C}{2}\sum_{i=1}^{N}e_{i}^{2},\;\;C>0$$

The constraint is as follows:

(4) $$y_{i}=w^{T}\phi \left(x_{i}\right)+\delta +e_{i}$$

where *e*_*i*_ is error variable, *e*_*i*_ ∊ *R*,* C* is regularization parameter, *C* > 0.

In solving the above optimization problem, the Lagrangian function is introduced as:

(5) $$L\left(w,\delta ,e;\alpha \right)=J\left(w,e\right)-\sum_{i=1}^{N}\alpha_{i}\left\{w^{T}\phi \left(x_{i}\right)+\delta +e_{i}-y_{i}\right\}$$

where *α*_*i*_ is the Lagrange multiplier, *α*_*i*_ ∊ *R*.

The optimization problem solved according to the KKT condition has the following solution:

(6) $$\begin{array}{c} \left(\begin{array}{cc} 0 & I^{T}\\ 1 & \Gamma +C^{-1}I \end{array}\right)\left(\begin{array}{c} \delta \\ \alpha \end{array}\right)=\left(\begin{array}{c} O\\ y \end{array}\right) \end{array}$$

where *y* = [*y*_1_, *y*_2_, ···, *y*_*N*_], *α* = [*α*_1_, *α*_2_, ···, *α*_*N*_], Γ_*ij*_ = *φ*(*x*_*i*_)^*T*^ *φ*(*x*_*j*_) = *k*(*x*_*i*_, *x*), *i*, *j* = 1, 2, ···, *N*,* k*(*x*,* x*) is a kernel function, *I* is unit matrix.

In this paper, the Gauss radial basis function (RBF) is used as a kernel function (Lu & Yang, 2007; Zhu, Ling, Wang, Hao, & Ding, 2018). After obtaining *δ* and *α* in Equation (6), *w* can be further calculated, and the nonlinear model obtained by LS‐SVM is as follows:

(7) $$y\left(x\right)=\sum_{i=1}^{N}\alpha_{i}\;\text{exp}\left\{-{||x-x_{i}\left|\right|}_{2}^{2}/\sigma^{2}\right\}+\delta$$

When solving the above equation, the kernel parameter *σ* and the regularization parameter *C* have a great influence on the model fitting accuracy and generalization ability, in order to achieve the prediction effect, the two variables need to be PSO optimized.

### 3.2 PSO‐based parameter optimization

The basic idea of the particle swarm algorithm is to find the optimal solution through information transmission and information sharing among individuals in a group (Gu, Zhao, & Wu, 2010; Yao, Cai, & Zhang, 2009). Assuming that in a D‐dimensional search space, population *X* = (*X*_1_, *X*_2_, ···, *X*_*n*_) consists of *n* particles, where the *i*‐th particle is represented as a D‐dimensional vector *X*_*i*_ = (*x*_*i*1_, *x*_*i*2_, ···, *x*_*iD*_)^T^ that is the position of the *i*‐th particle in the D‐dimensional search space. According to the objective function, the fitness value corresponding to each particle position *X*_*i*_ can be calculated, which represents the pros and cons of the particle. The optimal position of the *i*‐th particle is *P*_*i*_ = (*P*_*i*1_, *P*_*i*2_, ···, *P*_*i*D_)^T^, whose corresponding fitness value is called the individual optimal solution *P*_best,*i*_; the optimal position of the population is *P*_*g*_ = (*P*_*g*1_, *P*_*g*2_, …, *P*_*g*D_), whose corresponding fitness value is called the global optimal solution *G*_best,*i*_. The search speed of particle *i* is *V*_*i*_ = (*V*_*i*1_, *V*_*i*2_, ···, *V*_*i*D_)^T^, and the particle updates its speed and position through individual value and group extremum during the iterative process as follows:

(8) $$\begin{array}{cc} & V_{\mathrm{id}}=wV_{\mathrm{id}}+c_{1}r_{1}\left(P_{\mathrm{id}}-X_{\mathrm{id}}\right)+c_{2}r_{2}\left(P_{\mathrm{gd}}-X_{\mathrm{id}}\right)\\ & X_{\mathrm{id}}=X_{\mathrm{id}}+V_{\mathrm{id}} \end{array}$$

where *w* is the inertia weight, *d* = 1, 2, ···, *n*,* V*_*id*_ is the particle velocity, *c*_1_ and *c*_2_ are acceleration factors, *r*_1_ and *r*_2_ are random numbers distributed in the range of [0,1].

In order to prevent the blind search of particles, whose position and speed are limited to a certain interval [−*X*_max_, *X*_max_], [−*V*_max_, *V*_max_].

### 3.3 Establishment of LS‐SVM model based on PSO optimization

To sum up, the specific steps of the least squares support vector machine modeling based on PSO are as follows:

1. A set of {*C*,* σ*} is randomly generated to establish the LS‐SVM regression model. The particle swarm dimension is set to 2, the number of particles in each particle swarm is 20, the number of iterations is 150, *c*_1_ = 1.5, *c*_2_ = 1.7, and the regularization parameter *C* and the kernel parameter *σ* are selected within the optimization range of 0~2,000 and 0.01~100, respectively.
2. The average absolute percentage error is chosen as the fitness function of the PSO algorithm, whose expression is as follows:
  (9) $$e_{\mathrm{ma}}=\frac{1}{N}\sum_{i=1}^{N}\left|\frac{y_{i}-{\hat{y}}_{i}}{y_{i}}\right|$$
  where *y*_*i*_ and ${\hat{y}}_{i}$ are the actual value and model prediction value, respectively, and *N* is the total number of training data.
  According to the size of each particle value, {*C*,* σ*} is substituted into the LS‐SVM reconstruction regression model, and the corresponding fitness value of each particle can be obtained from Equation (9) through the calculation results of the calibration sample.
3. According to calculating the fitness value of each particle, which is compared with the fitness value of individual optimal solution *P*_best,*i*_ and global optimal solution *G*_best,*i*_. If it is better than *P*_best,*i*_ and *G*_best,*i*_, update *P*_best,*i*_ and *G*_best,*i*_, otherwise keep the original data.
4. According to the PSO optimization Equations (8) and (9), the velocity and position of the particles are adjusted to produce new species.
5. Check the end condition. If the condition is satisfied, the optimization is ended; otherwise, go to step (3) until the maximum number of iterations is satisfied.
6. The LS‐SVM is assigned to the {*C*,* σ*} obtained after the optimization is completed. The prediction model is established by using the test data, whose prediction result of the test sample is obtained.

The LS‐SVM model that has been optimized is linearized at the sampling point *x*_0_ by using the Taylor formula, and the linearization model can be obtained through the method as follows:

(10) $$A\left(z^{-1}\right)y\left(t\right)=B\left(z^{-1}\right)u\left(t-1\right)+\partial$$

where *A*(*z* ^−1^) = 1 + *a*_1_ *z* ^−1^ + ··· + *a*_*n*_ *z* ^−*n*^, *B*(*z* ^−1^) = 1 + *b*_1_ *z* ^−1^ + ··· + *b*_m_ *z* ^−m^, *∂* is a constant.

## 4 GENERALIZED PREDICTION ALGORITHM FOR BACTERIA CONCENTRATION

After a simple model transformation, the constant ∂  is discretized, and the following controlled autoregressive integral moving (CARIMA) average model is obtained as follows:

(11) $$A\left(z^{-1}\right)y\left(t\right)=B\left(z^{-1}\right)u\left(t-1\right)+\frac{\varepsilon (t)}{\Delta }$$

where Δ = 1−*z* ^−1^ is a difference operator, *ɛ*(*t*) is an unrelated random sequence that represents the effect of random noise, and the discretized constant *∂* is contained in the random sequence *ɛ*(*t*).

After continuing processing according to the standard GPC method (Deng, Huang, Fei, Zhen, & Jiang, 2014; Guo, Chen, Zhu, & Hu, 2002), the multi‐step prediction vector expression that can output the predicted value is as follows:

(12) $$\hat{Y}=\text{GU}+F$$

where $\hat{Y}={\left[\hat{y}\left(t+1|t\right),\cdots ,\hat{y}\left(t+P|t\right)\right]}^{T}$ is the forecast output, *U* = [Δ*u*(*t*), ···, Δ*u*(*t* + *L*−1)]^T^ is the control increment, *F* = [*f*_1_(*t*), *f*_2_(*t*), ···, *f*_*P*_(*t*)]^T^ is a vector consisted of the free phases in the output prediction sequence, $G=\left[\begin{array}{ccccc} g_{0} & 0 & 0 & \cdots & 0\\ g_{1} & g_{0} & 0 & \cdots & 0\\ \vdots & \vdots & \vdots & \ddots & \vdots \\ g_{L-1} & g_{L-2} & g_{L-3} & \cdots & 0\\ \vdots & \vdots & \vdots & \vdots & \vdots \\ g_{P-1} & g_{P-2} & g_{P-3} & \cdots & g_{P-L} \end{array}\right]$ is the unit step coefficient, *P* is the prediction time domain, and *L* is the control time domain.

The moving horizon optimization performance index at *t* time in GPC takes the following form:

(13) $$\text{min}\;J=E\left\{\sum_{j=N_{1}}^{N_{2}}{\left[w\left(t+j\right)-y\left(t+j\right)\right]}^{2}+\sum_{j=1}^{L}\lambda \left(j\right)\Delta u^{2}\left(t+j-1\right)\right\}$$

where *E* is the mathematical expectation, *w* is the expected reference value of the object output, *N*_1_ and *N*_2_ are the initial and final values of the optimization time domain, respectively. *λ*(*j*) is a control weighting coefficient that is zero or a very small number, which can be increased until a satisfactory control effect is obtained if the control system is stable, but the control variable changes greatly in the actual selection (Liu, 2007.). The parameter *λ*(*j*) is generally set as a constant *λ*.

The reference trajectory is introduced to track it well for the output value:

(14) $$w\left(t+j\right)=\beta w\left(t+j-1\right)+\left(1-\beta \right)\cdot y_{s},\;\;j=1,2,\cdots ,N$$

(15) $$w\left(t\right)=y_{r}\left(t\right)$$

where *β* is the adjustment factor in interval [0, 1), *y*_*r*_ is the reference trajectory, and *y*_*s*_ is the set value for next moment.

When *W* = [*w*(*t* + 1), ···, *w*(*t* + *P*)]^T^, the formula (13) can be expressed as:

(16) $$J={\left(Y-W\right)}^{T}\left(Y-W\right)+\lambda \Delta U^{T}\Delta U$$

When $\frac{\partial J}{\partial U}=0$, the control amount can be obtained as follows:

(17) $$u\left(t\right)=u\left(t-1\right)+d^{T}\left(W-F\right)$$

where *d* ^T^ is the first line of (*G* ^T^ *G* + *λI*)^−1^ *G* ^T^.

**Figure 1:**
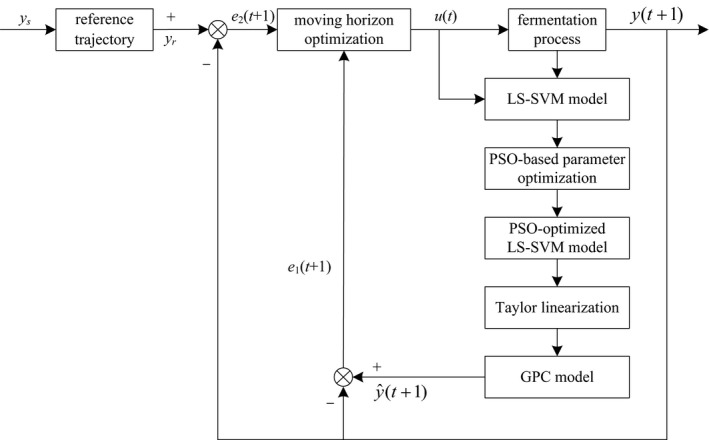
Generalized predictive control block diagram of lysozyme bacteria concentration based on least squares support vector machine (LS‐SVM)

The generalized predictive control block diagram of marine lysozyme bacteria concentration based on LS‐VM is shown in Figure 1.

## 5 TEST AND RESULT ANALYSIS

**Figure 2:**
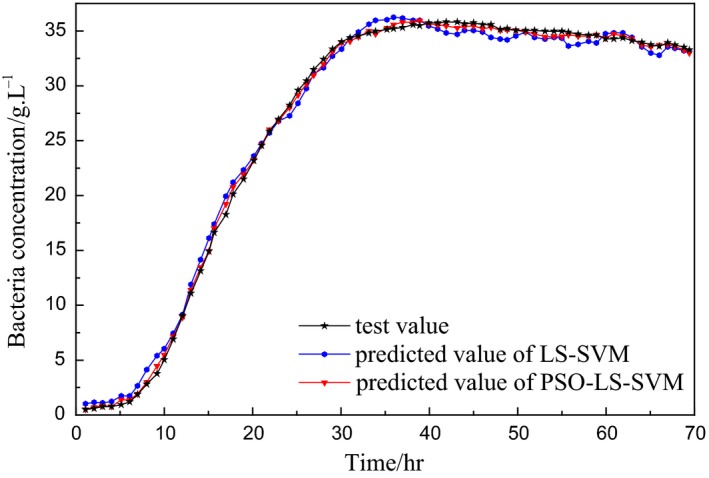
Simulation result of bacteria concentration

**Figure 3:**
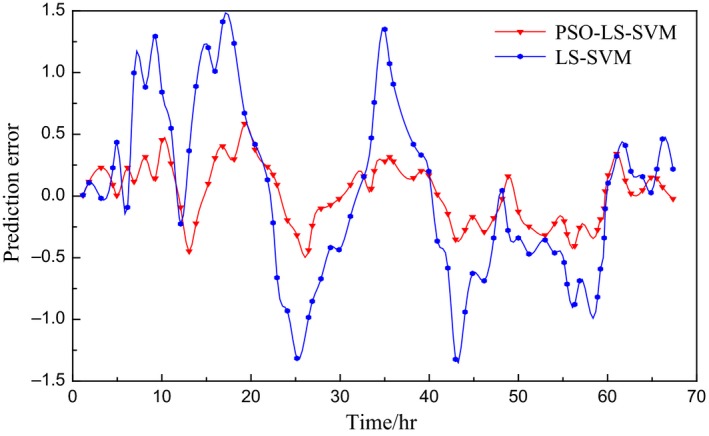
Prediction error curve of bacteria concentration

The experimental data are from the fermentation control system platform of Jiangsu University. The fermenter model is RT‐100L‐Y, and the fermented variety is lysozyme. Batch fermentation experiments are performed according to the medium formulation provided by the fermentation process. After high‐temperature sterilization of the fermenter steam, the tank pressure is controlled at 0.04 MPa by adjusting the gas output, the temperature is set at 32°C, the stirring speed is 400 *r*/min, the dissolved oxygen range is 35%–40%, and the pH is set at 7.3. In the experimental fermentation conditions, the control system collects the data of the substrate feed rate *f* that is obtained by the flow meter every hour and transmits it from the lower computer to the upper computer to form a database (Zhu et al., 2010). Under normal fermentation conditions, the bacteria concentration is measured by dry weight method. The fermentation broth is centrifuged at 20 ml/hr, washed with distilled water, and centrifuged twice; then, it is transferred to a constant‐weight measuring flask, dried to constant weight at 105°C, and weighed; the bacteria concentration (g/L) can be calculated (Sun, Wang, Huang, & Ji, 2010). According to the data collected by the upper computer, a batch of data is taken from one fermentation cycle, and 10 batches of data are extracted. The first nine batches of data are used as the training sample set, and the last batch is used as a test set. The simulation results are shown in Figures 2 and 3.

In the comparison of the prediction models in Figures 2 and 3, the LS‐SVM prediction model based on PSO optimization is obviously better than LS‐SVM model in fitting degree and prediction precision and has good modeling ability. Where the optimized parameters after PSO optimization are *C *=* *508.06 and *σ* = 8.32.

After data preprocessing, the modeling method introduced in this paper is used to train the data, which is verified the fitting degree and prediction accuracy with test data, and select the root mean square error (RMSE) and maximum absolute error (MAXE) as the evaluation criteria for model prediction accuracy.

(18) $$\text{RMSE}=\sqrt{\frac{1}{N}\sum_{i=1}^{N}\left(y_{i}-{\hat{y}}_{i}\right)}$$

(19) $$\text{MAXE}=\max_{i=1}^{N}\left|y_{i}-{\hat{y}}_{i}\right|$$

where *y*_*i*_ and ${\hat{y}}_{i}$ are the actual value and model prediction value, respectively, and *N* is the total number of training data. Two types of model simulation results are shown in Table 1.

**Table 1:** Performance contrast between PSO‐LS‐SVM and LS‐SVM

Prediction model	Bacteria concentration	
	RMSE	MAXE
PSO‐LS‐SVM model	0.1032	0.486
LS‐SVM model	0.7835	1.493

*Note*

LS‐SVM: least squares support vector machine; MAXE: maximum absolute error; PSO‐LS‐SVM: particle swarm optimization least squares support vector machine; RMSE: root mean square error.

By using the linearization method of this paper, the established LS‐SVM model is linearized and identified by Taylor:

(20) y(k)=0.673u(k)+0.109u(k−1)−0.435y(k−1)

where − 10 ≤ Δ*u* ≤ 10, 10 ≤ *u *≤ 55.

**Figure 4:**
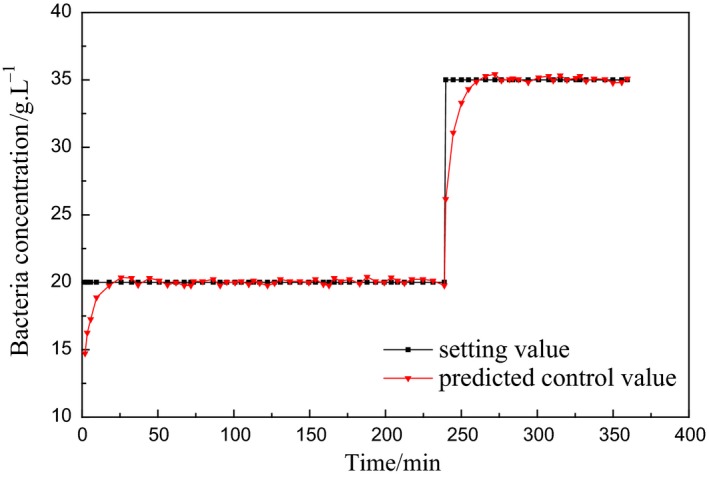
Simulation curve of bacteria concentration predictive control

**Figure 5:**
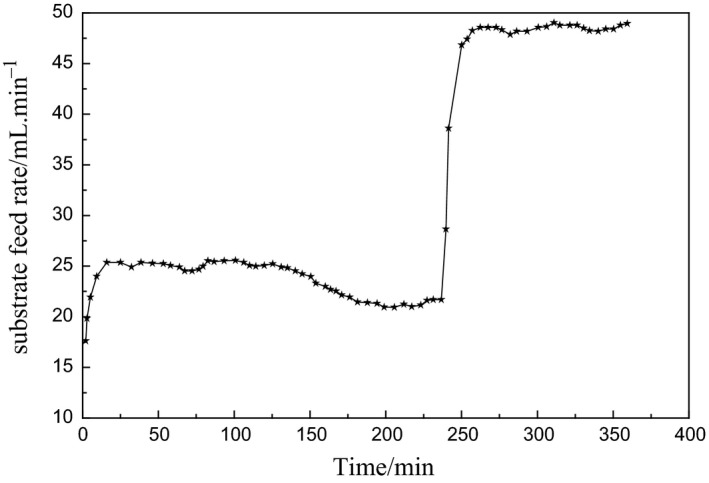
Controlled output simulation curve of substrate feed rate

During the accelerated and peak period of enzyme production, the cell increased logarithmically, and the activity and yield of the enzyme could be improved by controlling the bacteria concentration in this period. The predictive control is performed at the first 360 min of logarithmic growth period, where the prediction time domain is *P *=* *5, the control time domain is *N*_*u*_ = 3, the initial output is *u *=* *15, the initial increment is Δ*u* = 4.5, the initial output is *y *=* *14.5, and the simulation step length is 1 min. In the acceleration period of enzyme production, the bacteria concentration is not too high, which is set to 20 g/L; at the peak period of enzyme production, the growth rate of the bacteria is slowed down due to the rapid consumption of the substrate, which is set to 35 g/L to accelerate the substrate feeding rate and improve the enzyme activity and yield. The simulation results are shown in Figures 4 and 5.

From Figures 4 and 5, it can be seen that the LS‐SVM‐based predictive control outputs the better tracking reference trajectory and the output is relatively stable, and the error between predicted control value and set value is mostly at [−0.5, 0.5], the minority is at [‐1, 1]., which is consistent with the control requirements for the bacteria concentration in practical engineering.

**Figure 6:**
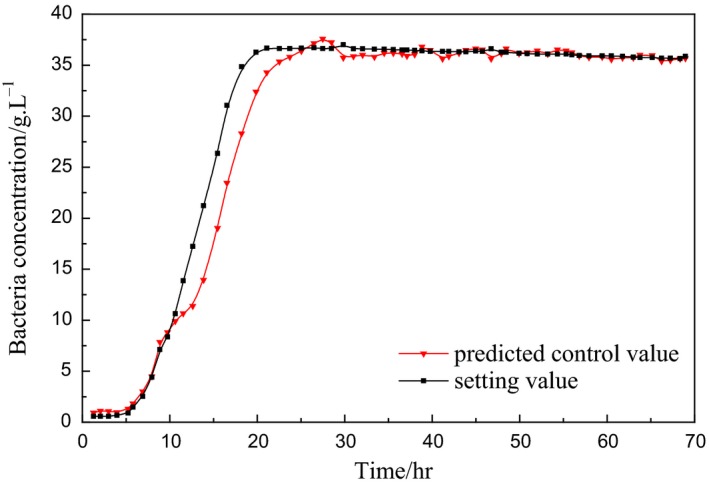
Experimental curve of bacteria concentration predictive control

**Figure 7:**
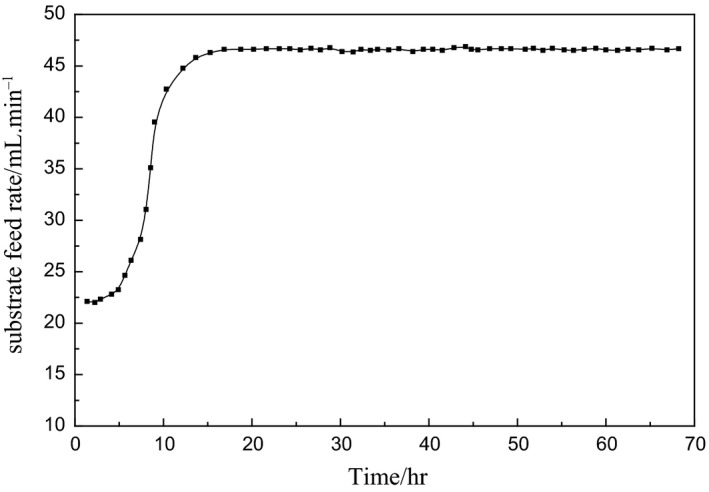
Controlled output experimental curve of substrate feed rate

Figures 4 and 5 are model‐based simulation results, so the input can be set as a step signal to study and analyze the performance of the control method. In the actual fermentation process, the bacterial concentration will not be changed rapidly, which is a slow time‐varying process, so the reference input also needs to be a relatively slow rising process. Figures 6 and 7 are control process diagrams of the actual fermentation process.

Under such control, the bacteria concentration grows fast during the growing phase and remains high during the producing phase, which is good for the enzyme productivity. The total activity of the enzyme is increased from 60% to 80%, and the yield is improved by 30% in the actual fermentation process.

## 6 CONCLUSION

In this paper, the generalized predictive control based on least squares support vector machine is proposed. After the regularization parameter *C* and kernel parameter *σ* of the model are optimized by using the particle swarm optimization algorithm, the LS‐SVM model of the bacterial concentration is established, which has high prediction accuracy and high fitting degree. To avoid solving nonlinear problems, the LS‐SVM model is linearized at each sampling point, and the generalized predictive control algorithm is used to solve the multi‐step prediction. The experimental results show that the method has good adaptability and robustness to the control of bacterial concentration in the fermentation process. It can be applied to the control of physicochemical parameters and biological indicators in the general fermentation process.

## CONFLICT OF INTEREST

The authors declare that they have no conflict of interests.

## ETHICAL STATEMENT

This study does not involve any human or animal testing.
